# Gilbert Syndrome in a Young Ethiopian Man: First Case Report

**DOI:** 10.4314/ejhs.v31i1.24

**Published:** 2021-01

**Authors:** Amir Sultan, Kibrewossen Kiflu

**Affiliations:** 1 Addis Ababa University, College of Health Sciences, Division of Gastroenterology & Hepatology, Addis Ababa, Ethiopia https://orcid.org/0000-0002-7000-9575; 2 Bethel Teaching Hospital, Addis Ababa, Ethiopia

**Keywords:** Gilbert Syndrome, Jaundice, Hyperbillirubinemia, Liver, Ethiopia

## Abstract

**Background:**

Gilbert syndrome is a well-recognized condition causing unconjugated hyperbilirubinemia with otherwise normal transaminases and liver function tests.

**Case:**

A 21 year old male patient presented with recurrent episodes of jaundice over four years. The episodes were preceded by stressful conditions and intercurrent illnesses. All laboratory prameters were normal except an unconjugated hyperbilirubinemia. A diagnosis of Gilbert syndrome was made after careful clinical evaluation.

**Conclusion:**

Recognizing Gilbert syndrome has important clinical implicaitions by avoiding uncessary and expensive workup of patients with jaundice. Mangement entails avoiding stressful conditions and prolonged fasting.

## Introduction

Jaundice is a major clinical symptom usually associated with liver diseases and hemolytic disorders. However, there are other well documented inhered causes of hyperbilirubinemia with varied clinical manifestations. Among these, Gilbert syndrome is a well-recognized condition causing unconjugated hyperbilirubinemia with otherwise normal transaminases and liver function tests (1). The condition has not yet been reported in Ethiopia, and below we present the first case report of the condition.

## Case

The patient is a 21-year-old male who presented to an internal medicine clinic in Addis Ababa in January 2020 with jaundice. The jaundice was preceded by a period of stress related to an exam he had a week earlier. He did not have vomiting, abdominal pain, diarrhea, bleeding or alteration of mental status

On detailed questioning, he reported that he had repeated episodes of jaundice over the last four years. On average, he was having 4 episodes of jaundice in a year and most of the time the episodes were associated with a period of stress and intercurrent illnesses. The last two episodes of jaundice were triggered by an upper respiratory tract infection and one followed an episode of mild dyspepsia.

On evaluation, he was noted to be in apparent healthy state. He was well built with fair complexion and had stable vital signs. He had normal abdominal examination, and there were no features of chronic liver disease in other organ systems.

He had undergone lab investigations during those episodes of jaundice. His CBC showed a hemoglobin of 16.8 gm/dl with an MCV of 87 fl. WBC count was normal and the platelet count was 233,000. There was no feature of hemolysis, and the LDH was 127 IU/l (normal range up to 248 IU/l). The Alanine Transaminase (ALT) was 17 IU/l and the Aspartate transaminase (AST) was 24 IU/L (normal up to 30 IU/l) and the alkaline phosphatase was 64 IU/l (normal up to 150 mg/dl). Both Hepatitis B surface antigen and Anti Hepatitis C antibody were negative. The total bilirubin was 4.4 mg/dl (Normal < 1.0 mg/dl), and from this, the conjugated (Direct) bilirubin fraction was only 0.2 mg/dl.

Based on these results and clinical presentation, the patient was diagnosed to have Gilbert's syndrome. As part of management he was advised to avoid stressful conditions and prolonged fasting.

## Discussion

Gilbert syndrome is a defect in bilirubin glucuronidation caused by a mutation in the upstream promoter part of the human chromosome number two (2). It is an autosomal recessive condition characterized by different variations of mutations. It is defined as benign, familial, mild, unconjugated hyperbilirubinemia (serum bilirubin 1–5 mg/dl) not due to hemolysis and with normal routine tests of liver function (3). It is a fairly prevalent condition in the world with a prevalence rate of 12.4% reported from Germany and 8% from Egypt (4). There are genetic variabilities to the prevalence of the condition that account for the varied epidemiology worldwide (5)

The most common presentation of the condition is jaundice in association with a stressful condition. Most commonly reported scenarios are fasting, hemolysis, intercurrent febrile illnesses, physical exertion, stress and menstruation (1). On the other hand, administration of corticosteroids and phenobarbitone improve the jaundice by increasing hepatic bilirubin uptake and by inducing hepatic conjugation enzymes respectively (3).

The condition tends to manifest after the period of adolescence because of alteration of the enzymes due to sex hormones. It is rare to diagnose Gilbert syndrome before puberty (3). Befitting to this, our patient also started to have the symptoms after the age of seventeen and was asymptomatic before then. The diagnosis of Gilbert syndrome is largely clinical; the important elements are unconjugated bilirubin in several occasions, a normal blood cell count and a normal liver enzymes level. Provocative testing such as prolonged fasting test and rifampin provocation test have been studied for diagnosis, but the tests are not applied clinically commonly (1).

Patients with Gilbert syndrome do not require specific forms of treatment due to the benign nature of the condition. The patients are advised to be cautious when using drugs like acetaminophen and irinotecan because of the potential exacerbation of the symptoms during treatment with such agents. However, the reported adverse events are not high compared to the prevalence of the condition. The quality of life of patients might be affected by the condition as the repeated episodes of jaundice could cause depression and impairment in social functioning (4).

The importance of recognition of Gilbert syndrome lies in the fact that these patients would have repeated episodes of jaundice, and extensive evaluation of the patients in such times is not cost effective. In general, as well as specialized medical practice in Ethiopia, jaundice is considered as an ominous symptom necessitating extensive workup. This should be practiced in most cases, but when a patient presents with such recurrent episodes, Gilbert syndrome should be considered and focused evaluation should be made. In addition, patients should be counseled in relation to their condition so that unnecessary cost could be avoided. The description of such a case from Ethiopia would be helpful in putting the condition as one good differential diagnosis in the evaluation of unconjugated hyperbilirubinemia.
